# Assessing English language sentences readability using machine learning models

**DOI:** 10.7717/peerj-cs.818

**Published:** 2022-01-04

**Authors:** Shazia Maqsood, Abdul Shahid, Muhammad Tanvir Afzal, Muhammad Roman, Zahid Khan, Zubair Nawaz, Muhammad Haris Aziz

**Affiliations:** 1 Institute of Computing, Kohat University of Science and Technology, Kohat, KPK, Pakistan; 2 NAMAL Institue of Mianwali, Mianwali, Punjab, Pakistan; 3 Robotics and Internet of Things Lab, Prince Sultan University, Riyadh, Saudi Arabia; 4 Department of Data Science, Faculty of Computing and Information Technology, University of the Punjab, Lahore, Punjab, Pakistan; 5 Mechanical Engineering Department, University of Sargodha, Sargodha, Sargodha, Punjab, Pakistan

**Keywords:** Sentence readability, Flesch-Kincaid, Language learning, Machine learning, Natural language processing

## Abstract

Readability is an active field of research in the late nineteenth century and vigorously persuaded to date. The recent boom in data-driven machine learning has created a viable path forward for readability classification and ranking. The evaluation of text readability is a time-honoured issue with even more relevance in today’s information-rich world. This paper addresses the task of readability assessment for the English language. Given the input sentences, the objective is to predict its level of readability, which corresponds to the level of literacy anticipated from the target readers. This readability aspect plays a crucial role in drafting and comprehending processes of English language learning. Selecting and presenting a suitable collection of sentences for English Language Learners may play a vital role in enhancing their learning curve. In this research, we have used 30,000 English sentences for experimentation. Additionally, they have been annotated into seven different readability levels using Flesch Kincaid. Later, various experiments were conducted using five Machine Learning algorithms, *i.e*., KNN, SVM, LR, NB, and ANN. The classification models render excellent and stable results. The ANN model obtained an F-score of 0.95% on the test set. The developed model may be used in education setup for tasks such as language learning, assessing the reading and writing abilities of a learner.

## Introduction

English is considered the language of science (Drubin & Kellogg, 2012). The comprehension of the English language is very significant because English-speaking countries are the leaders in developing innovations and discoveries. Excellency in the English language helps users to harvest opportunities from business to the entertainment industry. Further, focusing on the research literature often produces the English language, and thus, people worldwide study it as a second language. To cope up with this situation, English language courses are offered at various levels of education.

**Figure 1:**
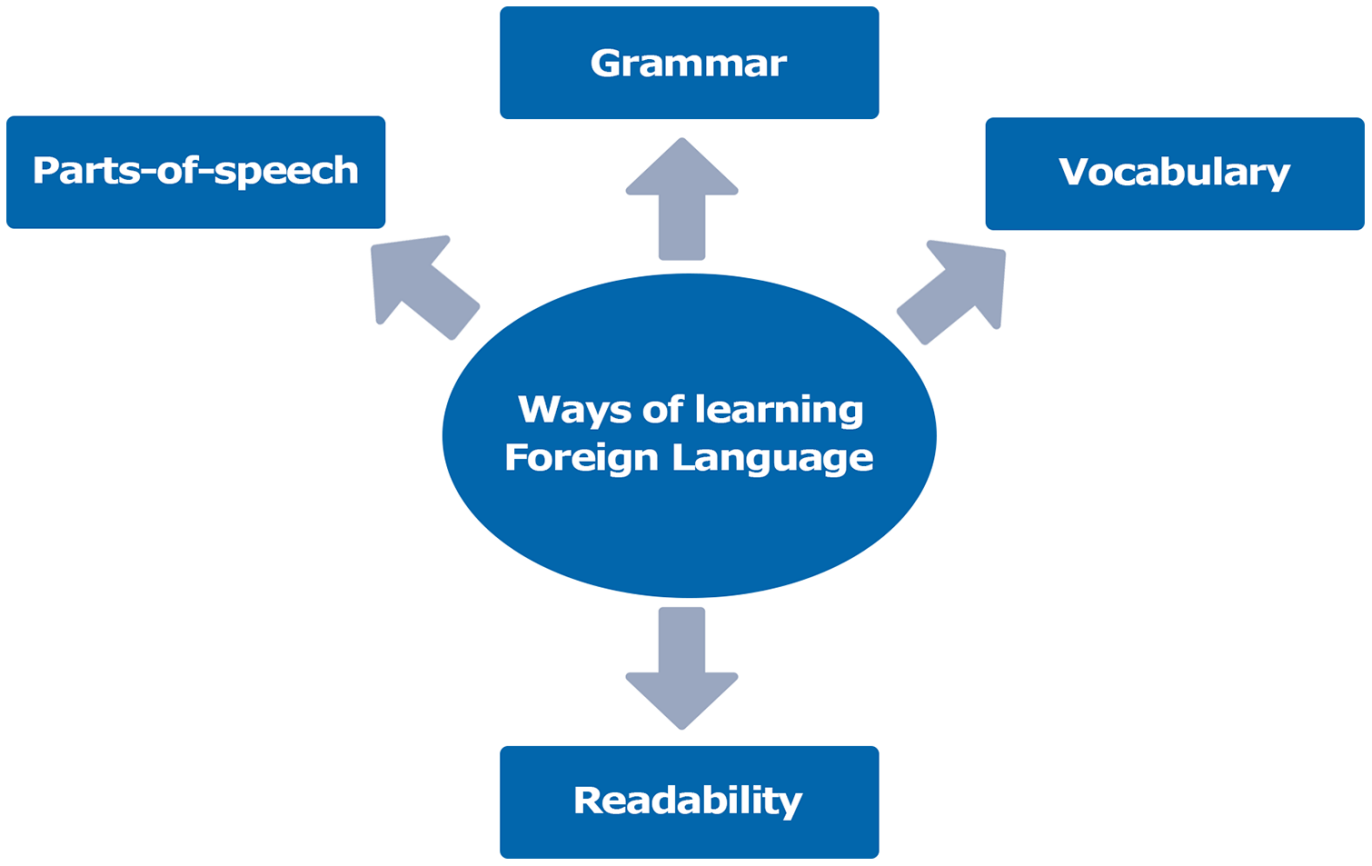
Ways of learning a foreign language.

Learning another language is always a tedious task. Any language can be understood by focusing on various aspects. These aspects are shown in Fig. 1. A Language may be learned by considering learning grammar, vocabulary, Parts-Of-Speech, and reading.

During the learning phase of a language, a learner may be interested in acquiring skills such as oral, writing, speaking, and reading. All these skills are dependent on understanding the constructs of the grammar of the language. Thus, the role of grammar in learning any language cannot be neglected. One may become aware of the language grammar while using that language for communication (Debata, 2013). And the same was reported by Hinkel and Fotos which say that a speaker can organize and express ideas in his mind with grammatical knowledge (Hinkel & Fotos, 2002). The vocabulary of a language is another essential part of learning a language. Various authors like Wilkins argue that vocabulary is the most essential part of learning any language because without vocabulary it is impossible to convey your message. (Wilkins, 1973). Parts-Of-Speech (POS) is another important cornerstone of a language. The POS helps a learner to properly use a word and it refines learner communication. Further, POS is the first step towards learning grammar and thus it is considered a natural choice for understanding a language. Language learning is not restricted to only these aspects; rather, there are some other aspects, such as readability. Reading is one of the most critical aspects of language learning. And to some authors, it is the most useful method in learning (Chi, Kuo & Peng, 2007). Further, Haynes, Huckin & Coady (1992), also consider that reading ability is also the main component of a second language in academic settings. Thus, without reading becoming proficient in a language is impossible.

Reading enables one’s comprehension, and indeed it is not an easy job. This can be validated by paying attention to the wide range of views of researchers about readability. For example, Carrol suggests that reading imitates a meaningful oral message from a text (Carroll, 1964). According to Grellet (1981), reading is the extraction of intended information from a text as efficiently as possible. This definition clearly describes that reading involves steps for constructing meanings from a given text. Similar to these definitions, several other researchers have defined readability terms in the same fashion, *e.g*., Koda (2004), Wolf (2007), and Smith (2004). The interest from so many researchers indicates that the reading process is an essential yet complex multifaceted process. When a teacher tries to improve students’ reading ability, they use reading exercises. To make this exercise useful, the texts must correspond to the student’s level (O’Connor et al., 2002). It means that there should be some measure of readability corresponding to the student’s level.

Readability measures demonstrate the ease with which a reader can understand a specific document. Chall & Dale (1995) defines readability as the “total number of elements in a given text that affect a reader’s success.” This reader’s success is a measure of understanding and reading at optimum speed. The creator of the SMOG readability formula (Simple Gobbledygook Mass), McLaughlin (1969), defines readability as “the level at which certain people find reading material convincing and understandable”. Pikulski (Maryansyah, 2016), on the other hand, suggests that readability is a measure of ease or difficulty with which a specific reader can understand a text material. All these varied definitions describe a general impression that it is related to the ease of reading. To be more precise on this topic, let's consider two definitions of the concept *Forestry*

1. **A scientific definition from *English Wikipedia***: “Forestry is the science and craft of creating, managing, using, conserving, and repairing forests, woodlands, and associated resources for human and environmental benefits.”
2. **A generic description from the Simple English Wikipedia: **“Forestry means working to take care of forests. Someone who has a job looking after forests is called a forester.”

The first statement provides more explicit content, but it is more sophisticated due to the complex sentence structure. The second definition of “*Forestry*” is more straightforward in terms of grammatical and document formats. From the reader’s point of view, the first interpretation concerns a more sophisticated audience, and the second is more appropriate for the public. Now the question is how we can automatically find the complexity of such kind of text?

Over the past 10 years, sophisticated NLP (Natural language processing) techniques such as syntactic parsing and statistical language modeling have been used to capture a text’s readability. The traditional readability formulas focus on a limited number of text features. These features are rough approximations of the linguistic factors contributing to the readability assessment. The purpose of the readability analysis is to assess the difficulty of an article for readers. The above example indicates that the difficulty of a given text is based on two factors: (1) the difficulty of words or phrases and (2) the syntax’s complexity (Collins-Thompson, 2014). For these factors’ characterization, the current work (Chall, 1958; Klare, 2000) is mainly based on features like average syllables per word, average words per sentence, *etc*.

In this paper, we presented the results of various machine learning-based models used to assess a given text's difficulty level. We predicted that the sentence readability measure for second language learners includes lexical, syntactic, and POS features that could perform better as a measure based on all of these features. The selected features are No-of-Words, No-of-Syllables, Noun-Phrase, Complex-words, Noun, Verb, Adverb, and Adjective. Further, we have used seven target classes describing the readability level of a given sentence. Finally, we conducted experiments on well-known techniques, *i.e*., KNN, SVM, LR, NB, and ANN. From the achieved results, we concluded that the ANN classifier achieved better performance results than the other classifiers with an F-means score of 0.95%.

The rest of this paper is structured as follows: First, the literature on measuring text readability is discussed. In the next section, the proposed methodology adopted for sentence complexity is explained. The details are discussed that how linguistic features were utilized and prepared for machine learning. In section four, we have presented the results achieved by performing different experiments to confer the research objective. Finally, the whole research work has been concluded, and future directions are provided.

## Related Work

Readability is one of the essential capabilities for learning a foreign language. In the literature, various researchers emphasize this aspect. For example, DuBay (2004) found more than 200 readability formulas exploiting linguistic, syntactic, and semantic clues for assessing the readability of a given text. It remained a matter of interest for researchers to know which text is more readable than others. We have categorized them as traditional and data-driven methods (machine learning-based).

### (A) Traditional methods for measuring readability

The readability formulas have long been used to help instructors to choose the suitable text for their students. One of them is the most well-known methods is Flesch’s and the formulas of Dale and Chall This is typical among the classical formulas, the first and far most important methodology paradigm developed in the field in the 1940s (Kincaid, 1975). This formula is simple, and its working is based on structural features such as total words, syllables, and sentence length. The Flesch formula is shown in Eq.1. It depends on the average sentence length (ASL) and the Number of Syllables per 100 Words (ASW).

(1) $${\rm LFlesch(d)} = 206.835 - 1.1015 \times ASL - 84.6 \times ASW$$

whereas

ASL = Average sentence length.

ASW = Number of syllables per 100 words.

Flesch–Kincaid Grade Level is a modification of Flesch’s Reading Ease Formula. It translates the former formula to U.S grade level. It is the most used formula that classifies texts into a grade level (Solnyshkina et al., 2017). Its generic form is shown in Eq. (2).

(2) $${\rm LFlesch(d)}= 0.39 \times ASL - 11.8 \times ASW - 15.59$$

whereas

ASL = Average sentence length.

ASW = Number of syllables per 100 words.

Similarly, the Gunning Fog index is also a measure of text readability based on the average number of words per sentence and the percentage of words with three or more syllables (Gunning, 1952). There exist a Fry readability measure, which is a readability metric for English texts. The Fry graph employs sentence length and number of syllables per 100 words. Fry plots these average numbers on Fry’s graph indicating the reading difficulty level (Rush, 1985; Danielson, 1987).

(3) $${\rm LFOG(d)}= 3.068 + 0.877 \times ASL + 0.984 \times MON$$

whereas

ASL = Average sentence length.

MON = complex words per total words

However, the researchers’ common consensus is that these structural features are not enough to describe complexity in their entirety. For example, they consider that longer sentences are grammatically more complex than shorter sentences may not always be true. Besides, word syllable count is used to infer that more frequent words have fewer syllables than less frequent ones (an association correlated with Zipf’s Law) (Kintsch & Vipond, 1979; Redish & Selzer, 1985). Again, syllable count doesn't need to represent the difficulty of every word. The current readability metrics are based on semantics. They usually make an approximation by frequency of words referring to a list or *corpus*. The Dale–Chall formula is dependent on average sentence length and the percentage of words. This formula does not consider the number of syllables per sentence. It checks if the words are in the list of Dall–Chall (list of 3,000 words) or not. It is given in Eq. (4). As it can be seen that it depends on ASL and the number of words found in Dale’s list.

(4) $$\text{LDale-Chall(d)}= \left( {0.1579 \times DS} \right) + \left( {0.0496 \times ASL} \right) + 3.6365$$

- ASL = Average sentence length.
- DS = The percentage of words not occurring on the Dale list of 3,000

Few other researchers like Roman et al. (2021) and Piergiovanni, Angelova & Ryoo (2020) also argued that these classical formulas consider only surface features, ignoring other important aspects that contribute to the text difficulties such as cohesion, coherence, and density of the text. In the 1980s, the second methodology paradigm *i.e*. structural-cognitive theories were used to compute text readability (Kintsch, 1979). Their work emphasizes utilizing higher text dimensions, such as inference loading, text density, and macrostructure. However, these efforts have not yielded better results than the classical approach, despite using more intricate features. Finally, the third type of work has recently emerged in this area, referred to as Artificial Intelligence (AI) based readability (François & Fairon, 2012). It contains three key features:

1. Using a large number of texts as training data.
2. Using an NLP function that can capture a broader range of readability factors.
3. Combining these features through a machine learning algorithm.

We have summarized the traditional readability formulas as shown in Table 1. These formulas are reproducible methods aimed at tailoring readers and texts to their level of difficulty. Details of various features used by the existing methods are grouped as “Element have taken into account.” It is self-explanatory that the first formula, *i.e*., Flesch-Kincaid, utilizes long word count, sentence count, word count.

**Table 1:** Traditional text readability methods.

		Elements have taken into account				
S. No.	Formula	Long word count	Sentence Count	Syllable Count	Word Count	Word frequency
1	Flesch-Kincaid (Flesch, 1948)	✓	✓		✓	
2	Flesch-Reading Ease (Flesch, 1979)	✓	✓		✓	
3	Gunning FOG Index (Gunning, 1952)		✓	✓	✓	
4	New Dale-chall (Chall & Dale, 1995)		✓		✓	✓
5	Fry Readability Graph (Fry, 1977)	✓	✓	✓	✓	

### (B) Data-driven methods

More indexes were developed in recent years that describes the complexity of sentence by considering additional complex features such as word frequency, word dictionary, text morphology, and the depth of parse tree. In our list, READ-IT is the first advanced tool for assessing the readability of the Italian language. The system is based on SVM (Support Vector Machine) to compute the complexity of the sentence. This system considers Lexical, syntactic, and Morpho-syntactic features to categorize the input text.

Apart from this Neural network (NN) model based on Long Short-Term Memory (LSTM) has been applied to measure the complexity of Italian sentences (Hochreiter & Schmidhuber, 1997). They have used lexical and syntactical aspects of the text. Apart from this, Recurrent Neural Networks (RNNs) based system is proposed to work based on NNs and analyze the data sequences (Lo Bosco, Pilato & Schicchi, 2018). Positions of words and punctuation are considered as sequences. Their proposed system assumes lemmas and syntactic structure to establish the complexity of the sentence.

Another system that measures sentence complexity is described in Bosco, Pilato & Schicchi (2018). It is also based on a recurrent neural network. The system measures the syntactic complexity of sentences written in the Italian language. The syntax of the sentence is expressed as a sequence of part-of-speech tags. The RNN learns the pattern and determines syntactic complexity, which is used in classifying Italian sentences based on their readability complexity.

Si & Callan (2001) and various other research studies predict text readability using the language model (Si & Callan, 2001; Collins-Thompson & Callan, 2004; Schwarm & Ostendorf, 2005; Yahya et al., 2019). Besides vocabulary, syntactic complexity is also an essential factor. Schwarm & Ostendorf (2005) and Heilman et al. (2007) have used syntactic features such as parse tree height for predicting reading grade level for readers. They also add another feature of entity coherence, which improves the overall classification accuracy. Si & Callan (2001) have used uni-gram models to classify science webpages. Recently, Collins-Thompson & Callan (2004) create a *corpus* by manually assembling the webpage to grade level. They observed that vocabulary words are not evenly distributed across the grade level. They made a “smoothed unigram” classifier to capture word usage variance according to grade levels. Their classifier outperformed some other difficulty measures and thus resulted in better quality predictions (Collins-Thompson & Callan, 2004). Schwarm & Ostendorf (2005) proposed an extended version of this approach. They used n-grams to capture the text’s syntactic complexity. They proposed combining statistical and traditional reading measures for assessing reading level. Their system has used four syntactic features: (1) average parse tree height, (2) the average number of noun phrases per sentence, (3) the average number of verb phrases per sentence, and (4) the average number of subordinate clauses per sentence (Schwarm & Ostendorf, 2005).

Petersen and Ostendorf later repeated and expanded Schwarm & Ostendorf (2005) work, which again confirms that classification and regression with SVMs provide a better reading proficiency approach at grade level (Petersen & Ostendorf, 2009). Another system that measures text readability is described in Pitler & Nenkova (2008). It includes a variety of linguistic factors. It combines lexical, syntactic, and discourse features, providing a highly predictive model for text readability judgment.

They made use of state-of-the-art machine learning models and NLP-based extracted features. Thus, they achieved significant performance gain and concluded that AI-based approaches might produce better results than their predecessors. In Table 2, we have critically evaluated each study, and analysis is given under various attributes. First column represent various research studies that predict text readability, Second attribute represents the methodology/algorithm that these studies utilizes. The system measures the complexity of sentences written in the different language is shown in column third. Fourth attribute is related with language dataset. Fifth and sixth column represent the benchmark and accuracy. Last column represent the features that system consider to categorize the input. As shown in Table 2, the comparative table compares existing systems based on their targeted aspect of language learning.

**Table 2:** Comparative analysis of machine learning methods measuring text readability.

Sr. No.	Research study	Methodology/Algorithm	Language	Language dataset	Benchmark	Acc	Elements have taken into account
1	READ-IT	SVM	Italian	Newspaper data/*corpus*	Flesch-Kincaid	80%	Lexical, syntactic feature
2	Si & Callan (2001)	Unigram model	English	educational Web pages	Flesch-Kincaid	75.4%	Surface linguistic feature and content feature
3	Schwarm & Ostendorf (2005)	SVM	English	An educational newspaper	Flesch-Kincaid and Lexile	Not mentioned	Syntactic feature, coherence
4	Pitler & Nenkova (2008)	Unigram model	English	Thirty articles from the Wall Street Journal *corpus*	Not mentioned	88%	Lexical, syntactic, and discourse feature
5	Heilman et al. (2007)	Unigram model	English	Textbook materials from English as a Second Language reading courses	Not mentioned	Not mentioned	Syntactic feature, coherence

The work summarized in Table 2 suggests that research on text classification by linguistic complexity suffers from the following limitations. It has been observed that previous studies on text readability are based on a traditional learning approach in which only a few lexical features are considered, and the essential features like syntactic feature and POS features are not considered. In-text classification, it isn’t easy to compare the effectiveness of various features belonging to different linguistic levels. The system mentioned above works on a small dataset and only uses few classifiers for text categorization. Further, the existing works look into the whole text rather than sentences.

Additionally, to the best of our knowledge, we argue that no single study was conducted on computing sentence readability of the English Language. One of the reasons for this could be that there is no publicly available dataset for this task. Therefore, this research aims at addressing these issues. We have also used a different machine and deep learning methods with additional linguistic features for predicting sentence readability.

## Study Framework

**Figure 2:**
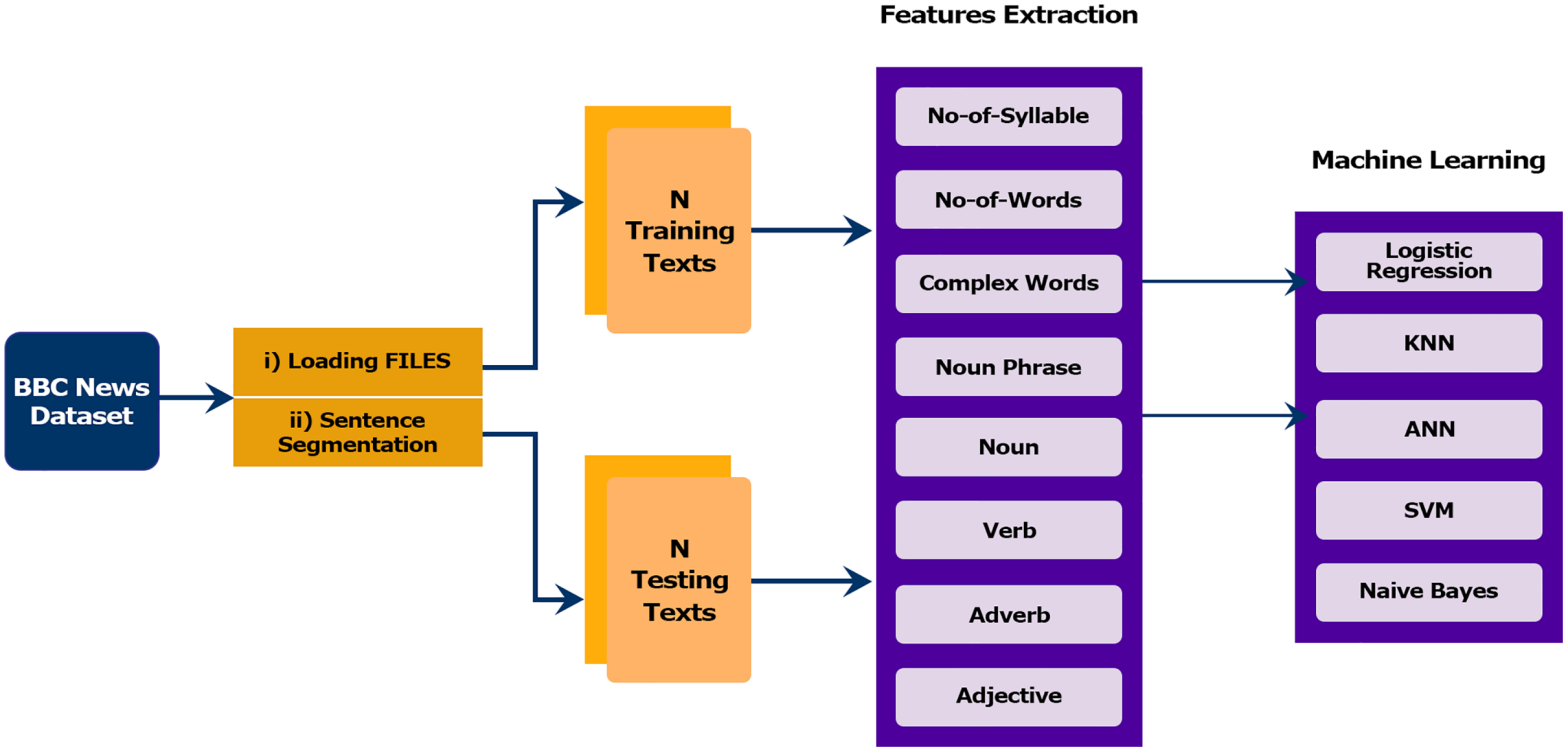
Proposed methodology adopted for sentence classification.

In this section, we describe the architecture of the proposed model that addresses finding the readability of sentences based on linguistic features. The proposed framework consists of four subcomponents: (1) Data collection and preprocessing, (2) Feature selection, (3) Classification modules and (4) Evaluation. The modules of the proposed method are presented in the framework outlined in Fig. 2.

Any data-driven solution depends on several important factors that affect the process.

The first factor is the choice of the dataset from which the model can be derived. The *corpus* should be divided into different levels of readability, where each section represents the readability level. Classification stability and reliability depend on the amount of data. Large amounts of data lead to a stable and reliable model.

The second factor is the algorithm. An effective classification algorithm is important to induce the model and classify the data. The algorithm must be proficient at handling multiclass classification.

The third most important factor is the selection of the features to be extracted from the data. The features should be good readability measurement. Therefore, features should be motivated by previous research to measure readability and also empirically evaluated.

### (A) Data collection preprocessing

To understand a sentence’s readability using a data-driven approach, it is a pre-requisite to have annotated dataset. We selected the BBC News *corpus*, which is compiled from BBC News in our previous work (Maqsood et al., 2020).

### (B) Features selection

Feature selection is one of the most significant processes in NLP. In this process, the most appropriate and suitable features are retrieved from the *corpus* to optimize the model’s quality and efficiency. The feature transforms the text into a vector space. In this paper, we have used many linguistics features for the evaluation of experimental results. The selected features were intended to provide an in-depth analysis of the sentences at different linguistic levels. In addition to traditional readability indicators, syntactic and lexical aspects have also been taken into account. Our feature set contained altogether eight features shown in Table 3. In Table 3, examples values for each attribute are mentioned in the last column for a sample sentence, *i.e*., “Some countries have tried to use fixed exchange rates.”

**Table 3:** Feature set.

S. No.	Features	Examples
1	No of Words	9
2	No of Syllables	13
3	Noun Phrase	1
4	Complex words	0
5	Noun	3
6	Verb	3
7	Adverb	0
8	Adjective	1

### (C) Sentence classification

With the growing increase of digital information, many articles describe the same topic of different readability levels. Therefore, the proposed work presents several machine learning models’ empirical results for evaluating text readability. The classifier techniques can build the model for text classification once the preprocessed features are retrieved. In this research, we have used those machine learning approaches that have been reported in NLP-related tasks. Of them, (LR, SVM, NB, and KNN) can be referred to as traditional machine learning algorithms, and deep learning approach (ANN) as advance/deep learning algorithms. In the following subsections, the detail of each classifier is illustrated.

### (1) Machine learning classifiers

In NLP, different machine and deep learning approaches have witnessed improved results, for example, sentiment analysis (Yang et al., 2016; Chauhan et al., 2020) and text categorization (Kusner et al., 2015). Machine learning approaches are used to build an automatic text classifier exploiting various features of documents. Thus, a classifier is made, and later it is used to classify the documents. The effectiveness of the developed modules is tested by applying it to the test dataset and checking the degree of correspondence between the classifier’s decisions. In assessing readability, the language models were introduced in the early 2000s (Collins-Thompson & Callan, 2004). Later, they were combined with classification algorithms such as LR, SVM, KNN, ANN, *etc*., to further increase accuracy (Petersen & Ostendorf, 2009).

### (a) Logistic regression

One of the earliest and widely known machine learning algorithms for classification method is LR (Peng, Lee & Ingersoll, 2002). This classification algorithm uses the sigmoid function and is usually used for the categorical type of variables. It has been reported that LR models are best suited for binary classification problems. However, it can be used on multi-class classification problems through the “one-vs-rest” method. In the One-To-Rest approach, the Logistic Regression creates a separate model for each class that takes one class as positive and the rest of all as negative. So, it trains *n* classifier for the data having *n*-classes in which for each class, it becomes a binary classification problem. In LR, the straight line is not directly fitted to the observation (*i.e*., data). Instead, the observation is based on the S-shaped curve known as the sigmoid. Using the logistic sigmoid function, the probability *P* is calculated by measuring the correlation between the categorical dependent variable and one or more independent variables, as shown in Eq. (5).

(5) $$P(x) =\frac{e^x}{e^x + 1},$$

where,

- *P*(*x*) is the output of the logistic regression model for a particular example.
- *y* = *w*_1_*x*_1_ + *w*_2_*x*_2_ +…+ *w*_n_*x*_n_+ *b*
  - The input features are represented by ‘*x*’.
  - The weight by ‘*w*’ and bias value by ‘*b*.’

If ‘y’ represents the output of the linear layer of a model trained with logistic regression, then sigmoid(y) generates the output to a discrete value of 0 or 1.

### (b) Support vector machine

Support Vector Machine (SVM) is a supervised learning and non-probabilistic algorithm (Cortes & Vapnik, 1995). This classification technique is based on the theory of statistical learning (Vapnik, 1998). Researchers have reported promising results in a different task using SVM. It supports binary classification by separating data points into two classes. The *One-To-Rest* approach can be used for multiclass classification by breaking down the multi-classification problem into multiple binary classification problems. SVM has been shown to work very well with high dimensional data and avoids the problem known as the dimension curse (Tan, Steinbach & Kumar, 2006). In machine learning, a dimension corresponds to the number of features in a feature space. The curse of dimensionality is to find the important feature amongst thousands of features. SVM uses vectors to represent the data. This establishes a straight-line boundary between vectors belonging to a particular class. Its mathematical representation is given in Eq. (6). In this equation, H represents the hyperplane, which is used to divide two classes through a line. And *w* is a weight, and *x* is an all-feature input matrix. *x1 … xn* represents per instance features, and *Y* is the output result. To classify test data, SVM requires the texts to be converted into vectors. We developed a method for assessing the readability level that uses support vector machines (SVMs) in our work. It considers many linguistic features used in the reading level assessment. Thus, our SVM model receives an input vector representing linguistic features. The SVM classifier views the data as points in a high-dimensional feature space. It aims to separate instances into classes with a hyperplane equivalent to a line in a two-dimensional space (Tanwani et al., 2009).

(6) $$\matrix{{\rm H}: w^Tx+b = 0 \\ {\rm Line}:\ Y = w_1x_1 + w_2x_2 +\ldots + w_nx_n + b }$$

### (c) Naïve Bayes

Naïve Bayes is based on the Bayes theorem probabilistic supervised learning model (Lewis, 1998). It is a popular and widely used machine learning method for text analytics tasks. In these models, individual words and categories’ joint probabilities are computed to predict a class for a given document. Its generic form is shown in Eq. (7), where *P*(*c*|*d*) is a conditional probability of a particular class sentence. In contrast, the prior probability is *P*(*c*), and the probability of observing x is *P*(*d*).

(7) $$P(c|d)={\frac{P(d|c)P( c )} {P(d)}}$$

Applying the independence assumption

$P(d{|c})= P(d_1|c) \times P(d_2|c)\times \ldots \times P(d_n|c)\times P(c)$

Substituting the independence assumption, we derive the Posterior probability of class given a new instance D as

$P(c|D)=P(d_1|c)\times P(d_2|c)\times\ldots \times P(d_n|c)\times P(c)$

This method calculates the probability of text belonging to a particular readability level. This implies that NB methods operate by associating features and then compute a probability that a text belongs to a specific readability level. The model's performance is evaluated on the test phase, which is not considered during the training phase. Finally, n-Fold cross-validation (usually 10-fold) is used to measure precision, recall, and F1-Score.

### (d) K-nearest neighbor classification model

In this paper, another classification model that was used is *K-*Nearest Neighbor (KNN). KNN model is an item-based machine learning model proposed by Cover & Hart (1967). In literature, its usage has been reported for filtering and routing emails, identifying different languages, classifying genres, and determining a text's readability level. In those tasks, grammatical features are exploited by KNN. It predicts the test sample category according to a given number (k) training samples of a similar kind. The similarity among the items is computed with some distance measure, *e.g*., Euclidean distance. The generic form of the Euclidean distance is shown in Eq. (8). The similarity between *d*1 and *d*2 is between 0 and 1; lesser numbers correspond to lower similarity, whereas higher numbers correspond to higher similarity.

Yang & Liu (1999) used KNN to classify input documents. It allocates a category to a document based on the KNN classifier, which ranks the neighbors under the training samples and uses the k top-ranking neighbors to predict the input document categories.

(8) $${\rm E (d1, d2) =}\sqrt {\mathop \sum \limits_{i = 1}^n {{\left( {{w_2}i - {w_1}i} \right)}^2}}$$

For using KNN for document classification, first, we try to *m-*dimensional feature vectors for each document. Once the documents are converted into vectors, then the similarity of each neighbor to X is calculated. Each neighboring document similarity score is used as the weight of its categories, and the total of category weights over the k nearest neighbors is being used for category ranking. The similarity can be determined between the two document vectors. By Euclidean distance or the cosine, The Distance measure is indeed a measurement of the distance between two documents, the basic formula of the Euclidean distance between documents D1 (*w*_1_, *w*_2_,…*w*_n_) and D2 (*w*_1_, *w*_2_,…,*w*_n_) is shown in Eq. (8). Where D1 and D2 are the documents and we have bag of words word 1, word 2, word 3 and so on. *w*_1_, *w*_2_,…*w_n_* are the frequency of word i in document D1 and D2.

### (e) Artificial neural network

Artificial Neural Network is a mathematical-based approach usually used for numerical and categorical data for classification and prediction purposes (Maksimenko et al., 2018; Bengio, Simard & Frasconi, 1994). ANN’s general structure usually consists of three layers, *i.e*., (1) an input layer, (2) one or more hidden layers, and (3) an output layer. An ANN with more than one hidden layer is referred to as Deep Neural Network. Each layer consists of multiple nodes. On the input layers, these nodes usually correspond to the number of input features. The nodes in the input layers are connected to the number of nodes in the middle layer. There are some weights assigned on every connection from the input layer to the hidden layer and subsequently to the output layer. Later these weights are varied to achieve the optimal classification results. There are different strategies for fine-tuning the weights, such as back-propagation, counter propagation, a three-layer feed-forward neural network, *etc*. (Khan, Shahid & Afzal, 2018).

In our case, inputs are the sentences, and the outputs are the sentence categories. We used Rectified linear unit (ReLU) or sigmoid activation function to generate output at each layer (Shahid et al., 2019; Abid et al., 2018; Warsi et al., 2020). Due to RelU, the output ranges are between 0 and max [0, 1], the sigmoid is assigned to the output layer that produces a probability of output between 0 and 1.

In the first step, the features for the whole train dataset are processed by ANN in the training phase. After the training, the weights and bias values are optimized to find the minimum loss value using the gradient descent algorithm. Then we can use those weights and bias values to make predictions.

## Results and Evaluation

This section evaluates the effectiveness of different models used to classify the sentences based on their complexity level. Five Machine-Learning algorithms are adopted in this research work: LR, SVM, KNN, NB, and ANN. Using machine and deep learning classification techniques, we trained our model and evaluated each classifier's performance based on test data. The following five most popular evaluation indexes are adopted to assess performance: (a) Accuracy, (b) Precision, (c) Recall, (d) F1-Score, and (e) Receiver Operating Character Curve (ROC Curve).

**Figure 3:**
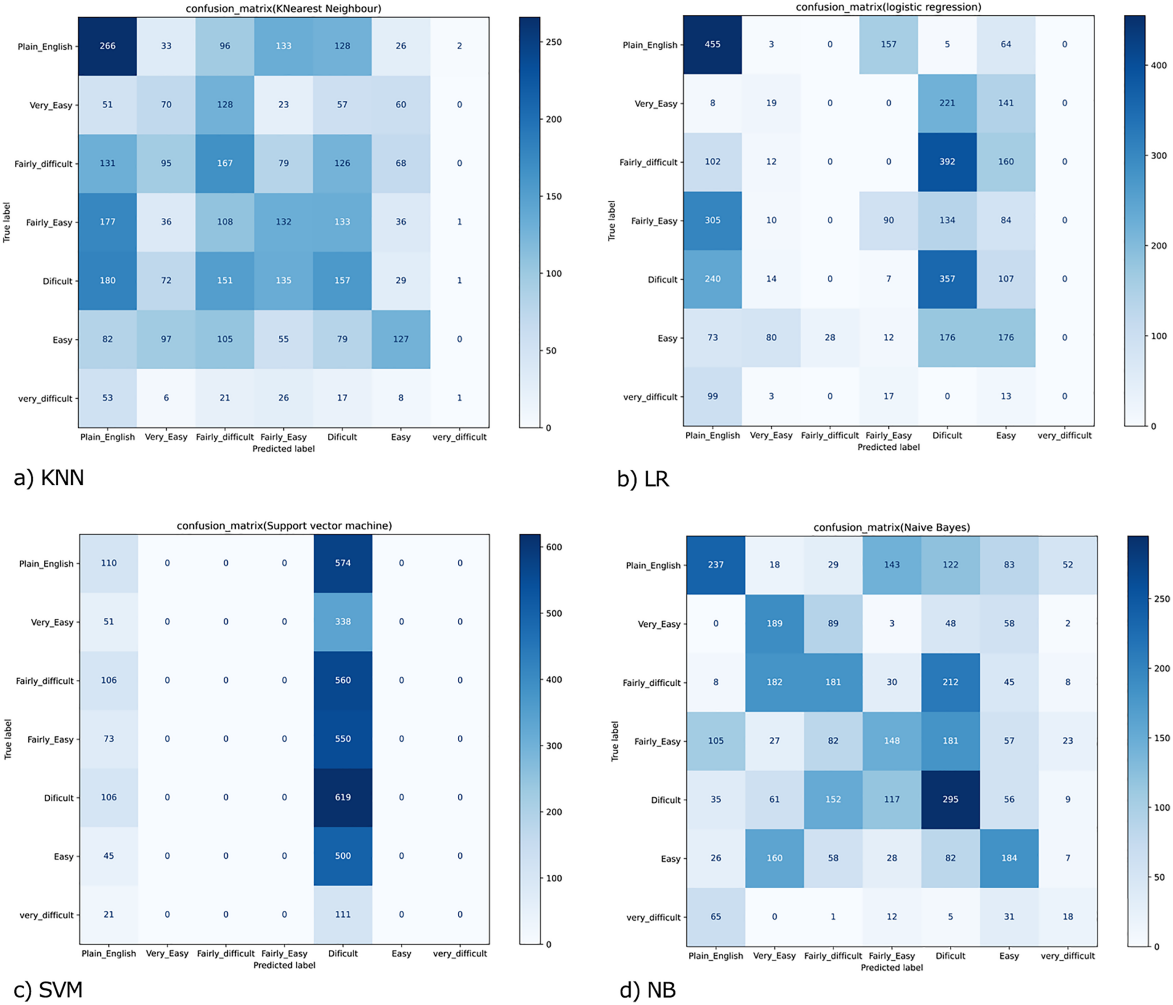
Confusion matrix for (A) KNN; (B) LR; (C) SVM and (D) NB.

The experiments were conducted exploiting all eight features shown in Table 3—this experiment insights into the five adopted ML (Machine learning) algorithms' performance. Figure 3 displays the cumulative measure of the performance for each classifier. Accuracy is the most classifying measure of performance. It is the calculation of all the actual groups correctly predicted per the total predictions. Accuracy, in particular, is a total score that refers to the correctly classified proportion of document or sentence belonging to seven different readability levels, either easy, fairly easy, or difficult-to-read, *etc*.

(9) $$Accuracy =\displaystyle{{correctly\; predicted\; class} \over {total\; testing\; class}}\times 100\%$$

Precision and recall have been computed for seven target readability levels: Precision, in particular, is the proportion of the number of a correctly labeled document or sentence over the total number of document or sentence classified by different machine learning classifiers as belonging to the different readability levels. The recall was determined as the ratio of the number of documents or sentences correctly categorized to the total number of documents or sentences in the test sets belonging to each reading standard.

(10) $$Precision = \frac{TP}{TP + FP}$$

(11) $$Recall =\frac{TP}{TP + FN}$$

TP is the number of specimens evaluated as positive. FP is the number of positive but negative testing samples, and FN is the number of negative but positive test specimens. The greater the precision and recall, the better the result displayed by the model. But, in some instances, the two are at odds with one another. The F-measure, which stands for the harmonic mean of precision and recall, is therefore proposed here. F1-Score are known as F measure and F score. It is a measure of test accuracy in problems under supervised learning (multi-class). F1 score is an average function of precision and recall. The mean precision and recall are measured *via* the F1 score.

(12) $$F\text{-}Measure ={\frac{2 \times Precision \times Recall} {Precision + Recall}}$$

However, the ROC (Receiver Operating Characteristics) curve is used to evaluate classifier output quality. It is one of the most significant assessment criteria to check the efficiency of any classification model. It provides a graphical representation of a classifier’s performance, rather than a single value like most other metrics

### (a) Traditional classifier results (KNN, LR, SVM, and NB)

Results for a traditional classifier can be visualized graphically by constructing a confusion matrix (Fig. 3). A confusion matrix is an effective approach to show the results of two or even more class classification problems. It simplifies the classifiers’ performance on test data and compares the classified data according to their actual class label. It demonstrates that the greater the accuracy, the better the model will predict the actual class based on the extracted features for each machine learning classifier. The confusion matrix for KNN, LR, SVM, and NB, are shown in (Figs. 3A–3D). We have seven classes, then our confusion matrix is a 7 × 7 matrix with the rows representing the true labels, and the predicted labels are shown in the columns. Both rows and columns are separated by a label, so the first row shows all the samples with a true label ‘Plain_English,’ and the last row shows all the samples with a true label ‘Very_difficult.’

Additionally, the diagonal element values reflect the degree of correctly predicted classes, as shown in Fig. 3A. The precision-recall metrics are specified in terms of the cells in the confusion matrix, specifically the use of the general term true positives and false negatives. In multiclass, precision is measured as the total of true positives amongst all classes divided by true positives and false positives. Using the confusion matrix shown in Fig. 3A  and considering the vertical axis values as the actual class and the horizontal axis values, the prediction. Then for the Class' Plain_English'.

- **True Positive (TP)** = 266 → samples of class ‘Plain_English’, classified as class ‘Plain_English’
- **False Positive (FP)** = 674 → (51 + 131 + 177 + 180 + 82 + 53) samples of classes ‘Very_Easy’, ‘Fairly_difficult’, ‘Fairly_Easy’,’ Difficult’, Easy’ and ‘Very_Difficult’, but classified as class ‘Plain_English’
- **False Negative** (FN) = 418 → (33 + 96 + 133 + 128 + 26 + 2) samples of class ‘Plain_English’, but classified as classes ‘Very_Easy’, ‘Fairly_difficult’, ‘Fairly_Easy’,’ Difficult’, Easy’ and ‘Very_Difficult’
- **True Negative** (TN) = 2406 → (70 + 128 + 23 … + 8 + 1) The sum of all the values in the matrix except those in column 1 and row 1

Similarly, we can also calculate the values for Very_Easy’, ‘Fairly_difficult,’ ‘Fairly_Easy,’’ Difficult,’ Easy,’ and ‘Very_Difficult’ classes, and thus the precision and recall values for KNN was achieved as 0.24 and 0.21.

**Figure 4:**
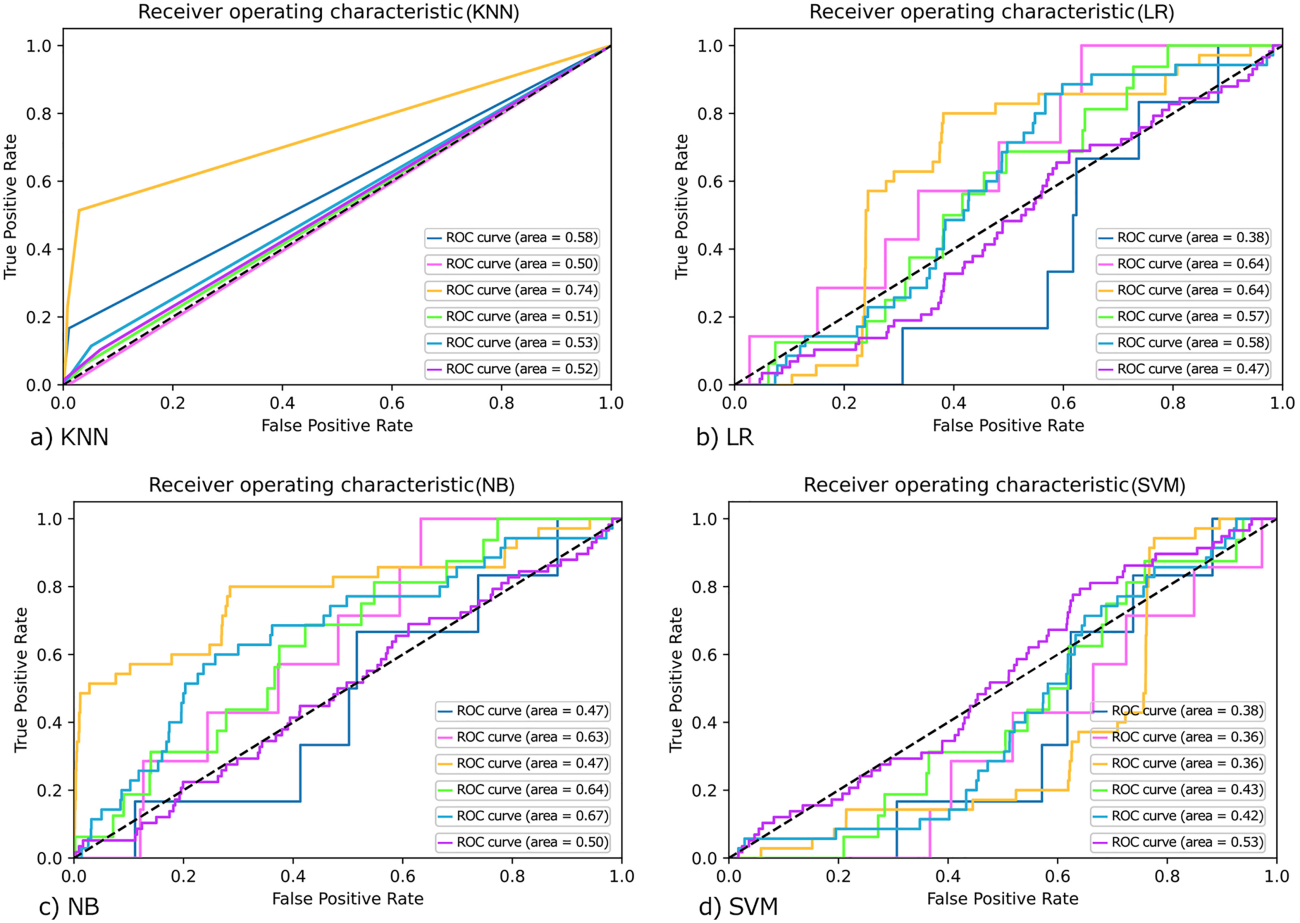
ROC curve for (A) KNN; (B) LR; (C) NB and (D) SVM.

Moreover, the ROC curve for KNN, LR, SVM, and NB are drawn as shown in Figs. 4A–4D). However, the ROC (Receiver Operating Characteristics) curve is used to evaluate classifier output quality. It is one of the most significant assessment criteria to check the efficiency of any classification model.

On the ROC graph, Classifiers may be evaluated by merely observing their location. In binary classification, ROC curves are usually used to study a classifier’s performance. For multi-class classification, the ROC curve is extended, and it is necessary to binarize the output. We can plot seven ROC curves in our work; one ROC curve can be drawn per label, as shown in Fig. 4. In which true positive values lie on the Y-axis and false-positive values are on the X-axis. We compare each algorithm’s classification performance on the synthetically set of data using the area under its ROC curves. Classifiers that give curves closer to the top-left corner indicate better performance. In Fig. 4A, a value of 0.5 for AUC (Area under curve) implies that the ROC curve lies on the diagonal, *i.e*., the curve gets closer and closer to the ROC's 45-degree diagonal space, the classifying data is less precise.

Similarly, the ROC curve for the rest of the classes was also generated, and they are shown in Figs. 4A–4D  as shown by the Figure that the ROC curve is near the diagonal. So, in this case, we can conclude that the system’s accuracy is not as far.

### (b) Deep learning algorithm results

**Figure 5:**
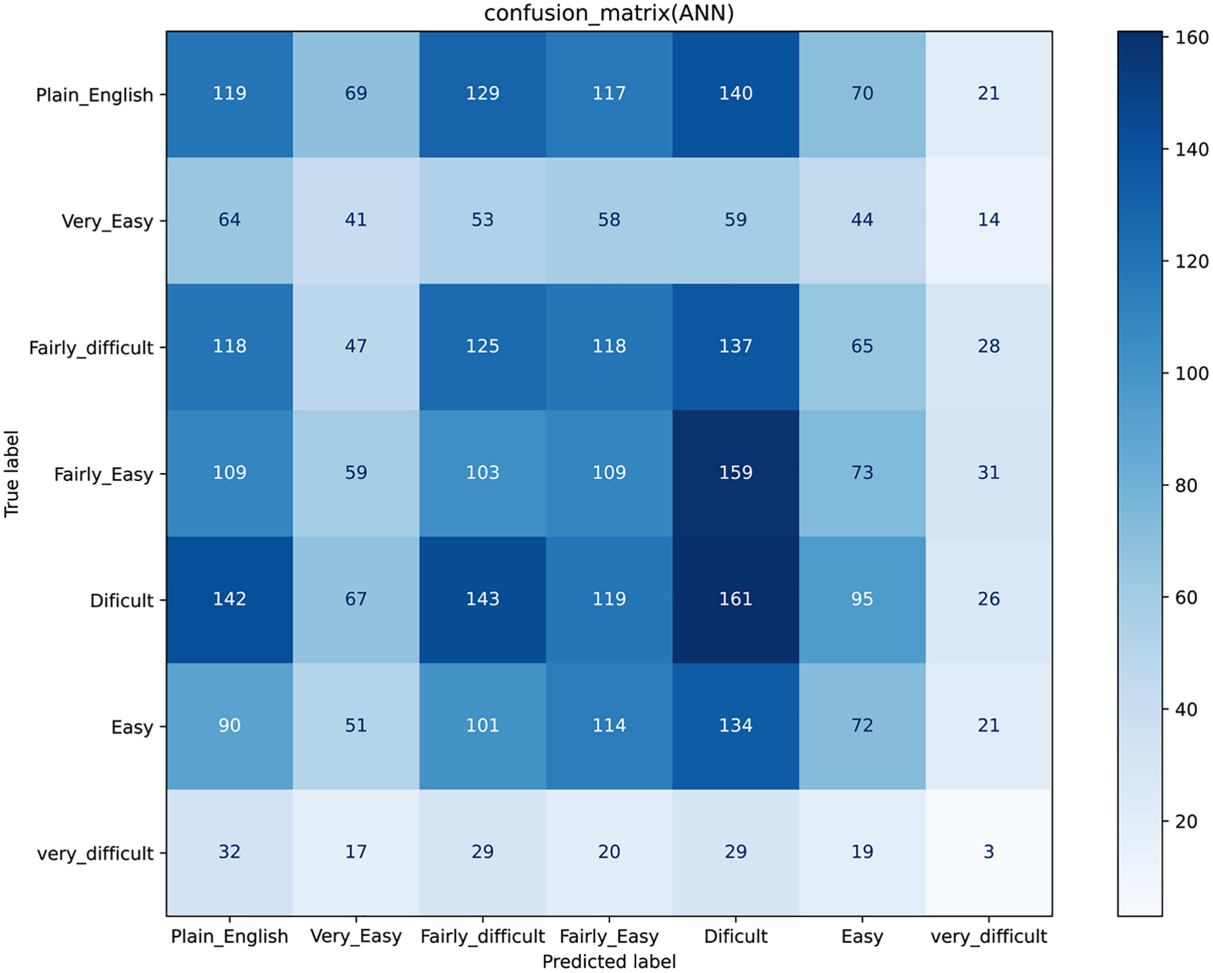
Confusion matrix for Artificial Neural Network.

**Figure 6:**
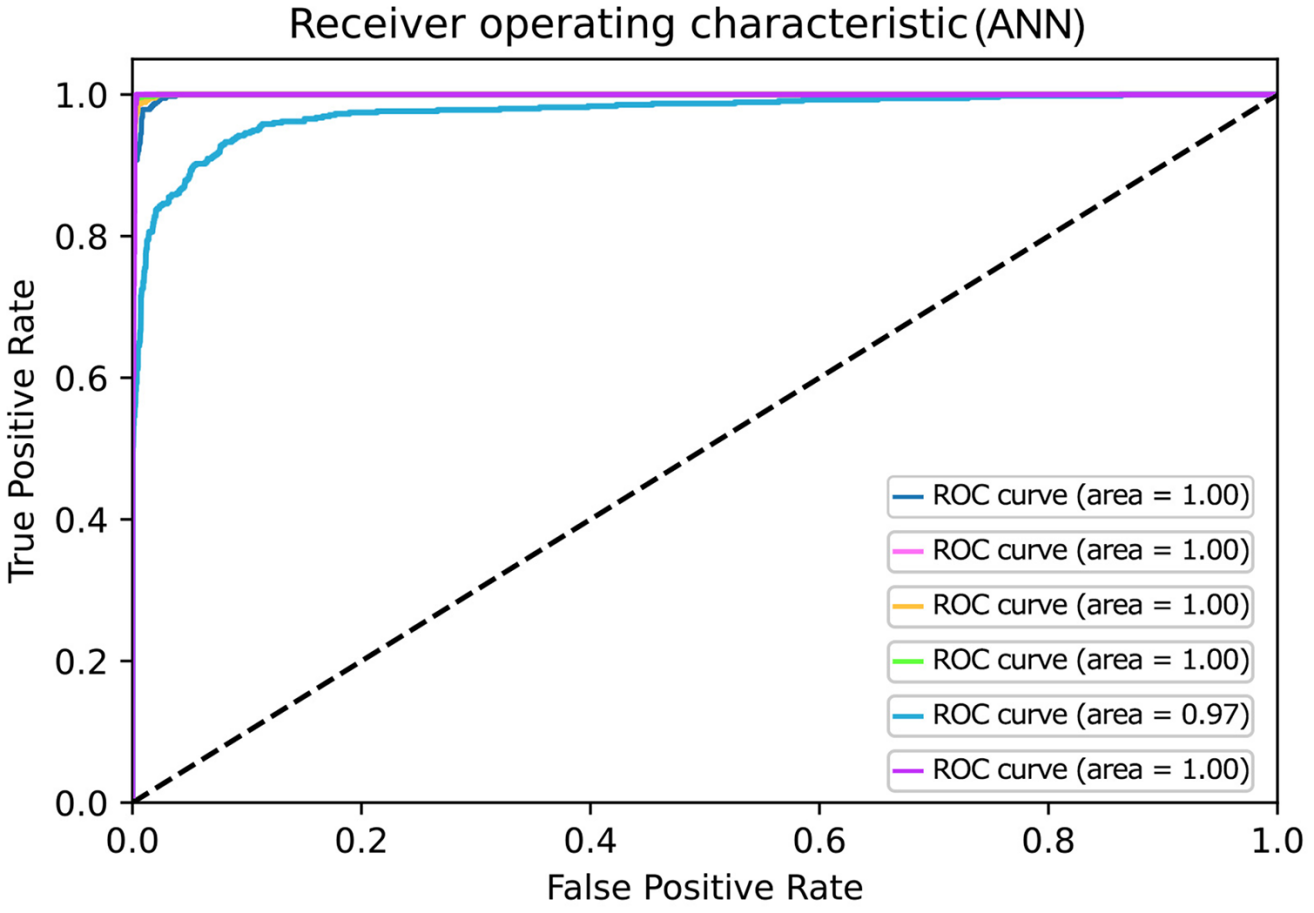
ROC curves for Artificial Neural Network.

An artificial neural network is a machine learning algorithm used for classification problems. The detail of which is discussed in the methodology section. This section shows the results for the Deep learning classifier, *i.e*., ANN, by visualizing graphically by constructing a confusion matrix and ROC curve as shown in Figs. 5 and 6. Figure 6 represents the ROC of the ideal classifier has AUC equal to 1 is perfectly accurate. This curve represents sensitivity and specificity of 100%, which is defined as below

**True Positive Rate** (**TPR**) is a recall generic term and is thus defined as follows:

(13) $${\rm TPR (Sensitivity)} =\displaystyle{{TP} \over {TP + FN}}$$

**False Positive Rate** (**FPR**) has the following definition:

(14) $${\rm FPR} (1\text{-}{\rm specificity) =}\frac{FP} {FP + TN}$$

In confusion matrices as mentioned above, we infer that the ANN model (Fig. 5) worked better than all other classification models (Fig. 3) by anticipating the relevant class labels and obtaining more significant performance outcomes with an accuracy of 0.96% than the different classifiers.

Similarly, ANN has also gained higher accuracy for ROC in contrast to other classifiers. Based on the confusion matrix and ROC curve plots results, it can be inferred that the ANN classifier obtained better performance results than other classifiers using different extracted features. As mentioned in the previous research area, ANN results compared with the traditional algorithm are best. In our case, ANN outperforms traditional algorithms. We believe that the results of the ANN are better due to its architecture as compared to the predecessors. The earlier used algorithms are statistical whereas the ANN is a nonlinear model that is easy to use and comprehend. Further, ANN is non-parametric where is the predecessors are parametric models that need a higher background of statistics. Additionally, ANN with a back-propagation learning algorithm is widely used in solving various classification and forecasting problems. Even though back-propagation convergence is slow, but it is guaranteed as was the case in our experiments as well.

**Figure 7:**
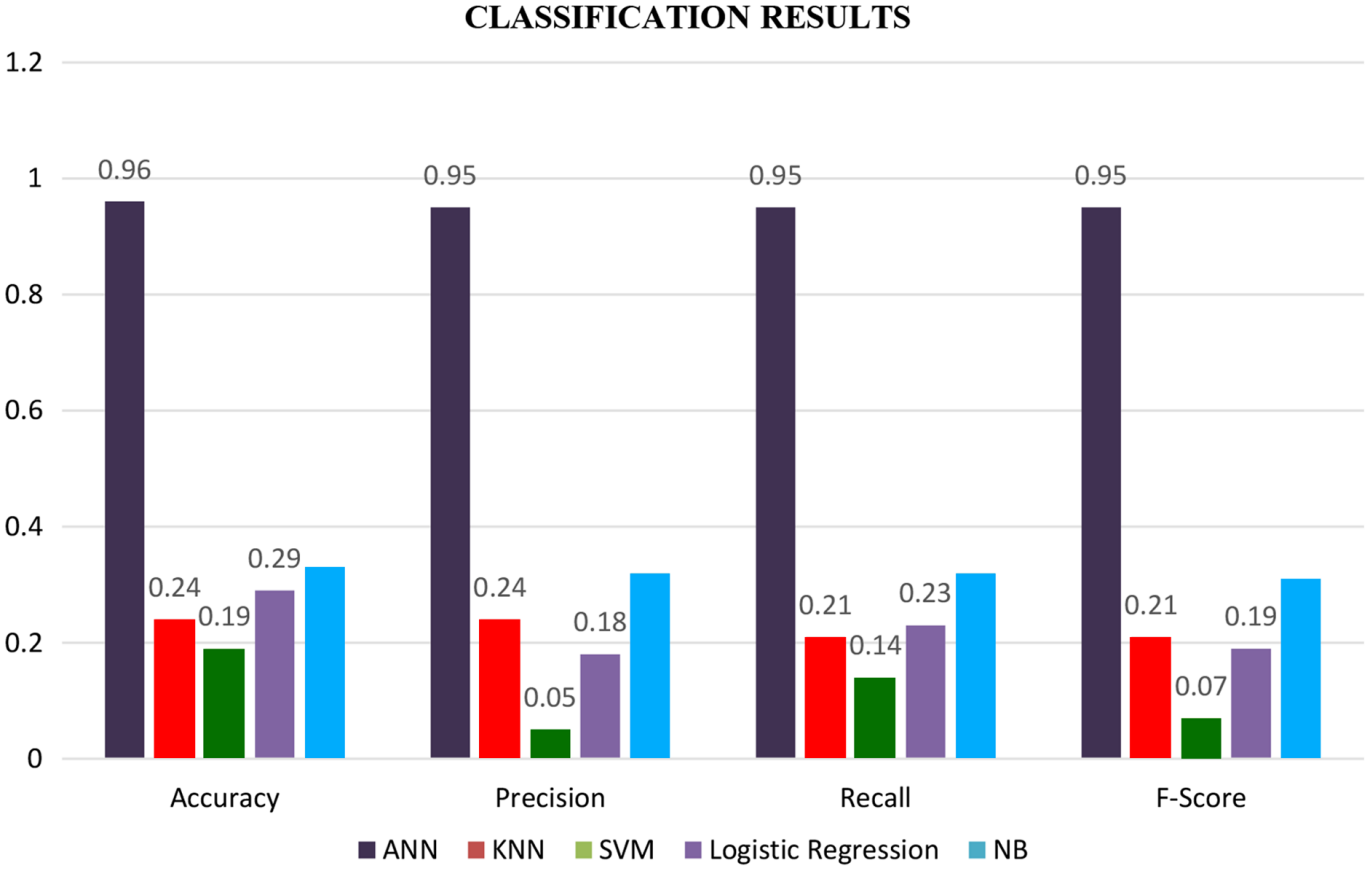
Classification results with all the features.

Finally, the results of all classifiers are combined in a graph shown by a bar graph as shown in Fig. 7. Where the brown bar represents ANN results, which are mentioned at the bottom of the graph. We see from the below-mentioned graph that ANN outperforms other classifiers in terms of accuracy, precision, recall, and F-score.

## Conclusion

English plays a central role in our educational system and national life. English is a language that has made a significant contribution to the advancement of learning. The role of English at a given time must influence both the way it is taught and the effect on the individual’s growth and everyday daily life. Learning a Foreign language increases the range of opportunities, from the business sector to entertainment. English is an integral part of the Higher Education system, and people all over the world study it as a second language. Therefore, English is being taught at different stages of education as an additional language. In English language learning, the readability aspect plays a crucial role in drafting and comprehending processes. This research provides basic work on the study of readability by developing models for classifying English sentences into readability levels. The problem has been targeted by training a machine and deep Learning classifiers, *i.e*., SVM, KNN, LR, NB, and ANN on features. The features are extracted from data representing seven different readability levels, *i.e*., Plain_English, very_easy, easy, difficult, very_difficult, fairly_difficuly, and fairly_easy. The classification results have been evaluated using standard text classification assessment techniques; namely, (a) Accuracy, (b) Precision, (c) Recall, (d) F1-Score, and (e) Receiver Operating Character Curve (ROC Curve). Experimental results showed that artificial neural networks (ANN) are better than other machine learning classifiers and provided good categorization performance as measured by accuracy and F1 score of 0.95%.

In the future, we plan to improve the results by exploiting more features. Further, we will try to determine which features are required to achieve an optimal classification rate.

## Supplemental Information

10.7717/peerj-cs.818/supp-1 Supplemental Information 1 Code used in the Project.

10.7717/peerj-cs.818/supp-2 Supplemental Information 2 Data set used in the Project.

## Additional Information and Declarations

### Data Availability

The following information was supplied regarding data availability: The data set and code are available in the Supplemental Files. They were retrieved from ML Resources: http://mlg.ucd.ie/datasets/bbc.html.
